# Dectin-1/Syk signaling triggers neuroinflammation after ischemic stroke in mice

**DOI:** 10.1186/s12974-019-1693-z

**Published:** 2020-01-11

**Authors:** Xin-Chun Ye, Qi Hao, Wei-Jing Ma, Qiu-Chen Zhao, Wei-Wei Wang, Han-Han Yin, Tao Zhang, Miao Wang, Kun Zan, Xin-Xin Yang, Zuo-Hui Zhang, Hong-Juan Shi, Jie Zu, Hafiz Khuram Raza, Xue-Ling Zhang, De-Qin Geng, Jin-Xia Hu, Gui-Yun Cui

**Affiliations:** 1 Institute of Stroke Center and Department of Neurology, The Affiliated Hospital of Xuzhou Medical University, Xuzhou Medical University, Xuzhou, People’s Republic of China; 2 Department of Neurology, Second Affiliated Hospital of Xuzhou Medical University, Xuzhou, Xuzhou, People’s Republic of China; 3 Department of Neurology, Drum Tower Hospital, Medical School of Nanjing University, Nanjing, People’s Republic of China; 4 Department of Rehabilitation Medicine, Linyi Cancer Hospital, Shandong, People’s Republic of China; 5 Department of Neurology, Suqian People’s Hospital of Nanjing Drum tower Hospital Group, Suqian, Jiangsu People’s Republic of China

**Keywords:** Dectin-1, Syk, inflammation, ischemic stroke

## Abstract

**Background:**

Dendritic cell-associated C-type lectin-1 (Dectin-1) receptor has been reported to be involved in neuroinflammation in Alzheimer’s disease and traumatic brain injury. The present study was designed to investigate the role of Dectin-1 and its downstream target spleen tyrosine kinase (Syk) in early brain injury after ischemic stroke using a focal cortex ischemic stroke model.

**Methods:**

Adult male C57BL/6 J mice were subjected to a cerebral focal ischemia model of ischemic stroke. The neurological score, adhesive removal test, and foot-fault test were evaluated on days 1, 3, 5, and 7 after ischemic stroke. Dectin-1, Syk, phosphorylated (p)-Syk, tumor necrosis factor-α (TNF-α), and inducible nitric oxide synthase (iNOS) expression was analyzed via western blotting in ischemic brain tissue after ischemic stroke and in BV2 microglial cells subjected to oxygen-glucose deprivation/reoxygenation (OGD/R) injury in vitro. The brain infarct volume and Iba1-positive cells were evaluated using Nissl’s and immunofluorescence staining, respectively. The Dectin-1 antagonist laminarin (LAM) and a selective inhibitor of Syk phosphorylation (piceatannol; PIC) were used for the intervention.

**Results:**

Dectin-1, Syk, and p-Syk expression was significantly enhanced on days 3, 5, and 7 and peaked on day 3 after ischemic stroke. The Dectin-1 antagonist LAM or Syk inhibitor PIC decreased the number of Iba1-positive cells and TNF-α and iNOS expression, decreased the brain infarct volume, and improved neurological functions on day 3 after ischemic stroke. In addition, the in vitro data revealed that Dectin-1, Syk, and p-Syk expression was increased following the 3-h OGD and 0, 3, and 6 h of reperfusion in BV2 microglial cells. LAM and PIC also decreased TNF-α and iNOS expression 3 h after OGD/R induction.

**Conclusion:**

Dectin-1/Syk signaling plays a crucial role in inflammatory activation after ischemic stroke, and further investigation of Dectin-1/Syk signaling in stroke is warranted.

## Introduction

In recent decades, ischemic stroke has become one of the most common causes of disability and mortality worldwide. Although the pathophysiology of cerebral ischemic injury is multifactorial, increasing studies have suggested that the inflammatory response plays a crucial role in stroke progression [1, 2]. Inflammation in the brain parenchyma after cerebral ischemia is mediated by neurons, endothelial cells, microglia, and other immune cells [3], and inhibition of inflammatory responses has been demonstrated to improve the outcome following a stroke [4, 5]. However, the detailed mechanisms by which the inflammatory response is triggered after a stroke remain largely unknown.

Dendritic cell (DC)-associated C-type lectin-1 (Dectin-1) has been identified as an immune-receptor tyrosine-based activation motif (ITAM)-coupled C-type lectin receptor (CLR). Dectin-1 recognizes various danger-associated molecular patterns (DAMPs) and triggers inflammatory signals by recruiting its downstream molecular tyrosine kinase (spleen tyrosine kinase; Syk) [6–8]. Dectin-1 is a type II transmembrane receptor that is expressed on different cell types during inflammation, including DCs, neutrophils, monocytes, T cells, and epithelial cells [9–12]. Previous studies have reported that immune receptors that initiate the inflammatory response play an important role in stroke progression [13–16]. However, to the best of our knowledge, whether the immune receptor Dectin-1 is involved in the inflammatory response following a stroke has not yet been investigated.

Syk is a nonreceptor protein tyrosine kinase that is observed extensively in hematopoietic and nonhematopoietic cells [17, 18]. It possesses tandem N-terminal src homology-2 (SH2) domains that can bind to ITAMs [19, 20]. Incomplete ITAMs, referred to as hemITAMs, can interact with Syk and mediate its activation [21]. A number of C-type lectin receptors, such as Dectin-1, C-type lectin-like receptor 2 (CLEC2), and C-type lectin domain-containing 9A (CLEC9A), possess hemITAMs and transduce signals through Syk [22]. Syk has been reported to play an indispensable role in acute and chronic inflammation, and inhibition of Syk impedes brain tissue damage following an ischemic stroke [23–26]. However, to the best of our knowledge, the specific role of Dectin-1 and its downstream target Syk in ischemic stroke has not yet been investigated.

**Fig. 1:**
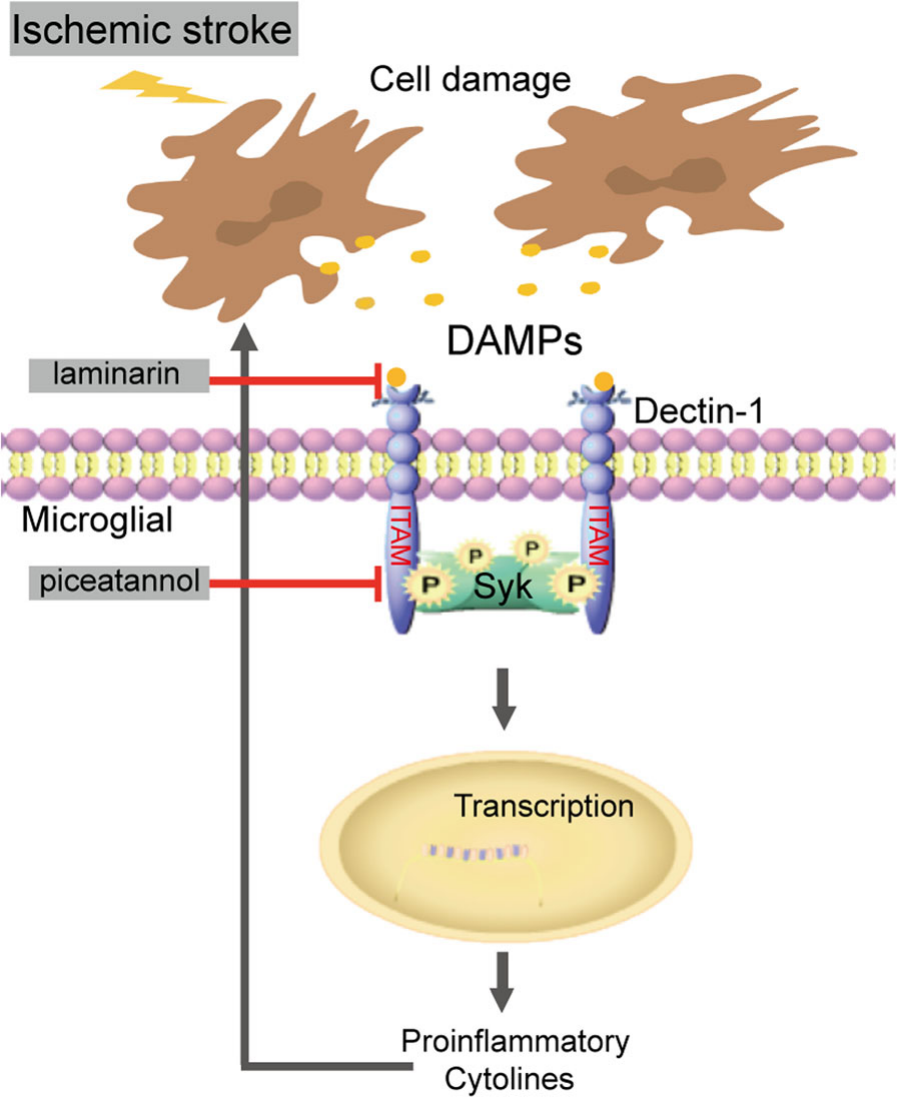
Working model for the present study. Injured cells release damage-associated molecular patterns and activate innate immunity via receptors, such as Dectin-1. Then, the ligand phosphorylates ITAM and recruits the downstream kinase Syk, which phosphorylates Syk and drives inflammatory cascades and cytokine production. The Dectin-1 antagonist laminarin and Syk inhibitor piceatannol can decrease the release of the inflammatory cytokines induced by ischemia. Dectin-1/Syk signaling appears to play a vital role in the inflammatory response after a stroke

The present study revealed that the Dectin-1/Syk pathway is involved in neuroinflammation following ischemic stroke. Changes in Dectin-1, Syk, p-Syk, TNF-α, and inducible nitric oxide synthase (iNOS) expression following ischemic stroke were evaluated both in vivo and in vitro. In addition, the present study investigated whether the Dectin-1 antagonist laminarin (LAM) or the Syk inhibitor piceatannol (PIC) could partly reverse neuroinflammation following ischemic stroke in mice. The working model for the present study is presented in Fig. 1.

## Materials and methods

### Cerebral focal ischemia

Adult male C57BL/6 J mice (weight, 22–30 g) were purchased from the Shanghai Experimental Animal Center of the Chinese Academy of Sciences (Shanghai, China). The cerebral focal ischemia model was induced by photothrombotic ischemia as previously described [27]. The main steps are as follows: The mice were injected with 1% Rose Bengal [100 mg/kg intraperitoneally (i.p.) dissolved in 0.9% saline] (Sigma-Aldrich; Merck KGaA) following induction of anesthesia with 10% chloral hydrate [300 mg/kg (i.p.) dissolved in 0.9% saline]. After 10 min, the sensorimotor region, which is located ~ 2 mm lateral to the bregma, was exposed for 15 min to cold light. In order to investigate the optimal ischemia time, the mice were made ischemic at 6 h and 1, 3, 5, and 7 days. The animals were randomly divided into the following four groups: (i) sham group, (ii) ischemic group with vehicle treatment (ischemia + saline/DMSO group), (iii) ischemia group, and (iv) Dectin-1 antagonist treatment group/Syk inhibitor treatment group (ischemia + LAM/PIC group).

### Neurological function tests

In all animals, the adhesive removal and foot-fault tests were performed and the modified neurological severity score (mNSS) was obtained before stroke and at 1, 3, 5, and 7 days after stroke with or without LAM/PIC treatment. The mNSS is a composite that is used to assess neurological functions based on motor, sensory, balance, and reflex measures, which are graded on a scale of 0 to 18 (normal score, 0; maximal deficit score, 18); higher scores imply greater neurological injury [28, 29].

### Analysis of the brain infarct volume

Formaldehyde-fixed specimens were cut into 50-μm-thick sections and subjected to Nissl’s staining. The sections were placed in 100, 95, and 80% ethanol for 30 s each and then treated with FD Cresyl Violet Solution^TM^ (FD Neoro Technologies) for 2 min. After washing in distilled water, the sections were dehydrated through an alcohol series, cleared in xylene, and coverslipped with neutral resin. In the present study, the stained sections were scanned and the infarct areas measured using the ImageJ software. The total infarct volume was obtained by indirect methods as previously described [27].

### Drug administration in vivo and in vitro

The Dectin-1 receptor antagonist LAM (Sigma-Aldrich; Merck KGaA) was diluted to 10 mg/ml in vehicle (saline) according to the manufacturer’s protocol. Then, LAM at 300 mg/kg/day or the same volume of vehicle (saline) was injected i.p. 1 h after ischemic stroke and once daily for the 2 subsequent days after the stroke. For the in vitro experiment, the optimal dose of LAM was also assessed (the Additional file 1). Then, BV2 cells were preincubated with LAM for 1 h before induction with oxygen-glucose deprivation/reoxygenation (OGD/R) or lipopolysaccharide (LPS; 1,000 ng/ml).

Similarly, the mice were treated with either the Syk inhibitor (PIC; Selleck Chemicals) or vehicle (DMSO) once daily after ischemic stroke. For the in vivo experiment, the PIC dose was 20 mg/kg/day. For the in vitro experiments, the optimal dose of PIC was also assessed (Additional file 1). Then, BV2 cells were pretreated with PIC for 1 h before treatment with or without OGD/R or LPS.

### Cell culture and experimental protocols

BV2 microglial cell line was grown in Dulbecco’s modified Eagle’s medium (DMEM)/high glucose supplemented with 10% fetal bovine serum, 100 U/ml of penicillin, and 100 mg/l of streptomycin at 37 °C in a 5% CO_2_ incubator. Mouse primary microglial cells were prepared as previously described [30, 31]. Briefly, brains were removed from mice at post-natal days 1–3 and the cortices were triturated into single cells. Mixed glial were plated in 25 T culture flasks coated with poly-d-lysine and grown in DMEM/F12 with 10% fetal bovine serum, GlutaMAX (Invitrogen; ThermoFisher Scientific, Inc.) and 1% penicillin/streptomycin in a 5% CO_2_/37 °C incubator, changing medium after 7 days, for a total of 2 weeks. In order to harvest primary microglial cells, the flask was shaken at 150 rpm for 2 h. The fluid medium was subsequently collected and centrifuged at 1,000 rpm for 10 min. The cell pellets were resuspended to plate 5 × 10^5^ cells per well onto 6-well plates, and subjected to various treatments within 24 h of harvest. Cells were pretreated with LAM or PIC for 1 h and then subjected to OGD/R or LPS treatment. For OGD/R, the cells were washed twice with phosphate-buffered saline (PBS), placed in glucose-free DMEM and then exposed to hypoxia (94% N_2_, 5% CO_2_, and 1% O_2_) at 37 °C in a humidified chamber (ThermoFisher Scientific, Inc.) for 3 h. Then, OGD was terminated, and the cells were exposed to normal culture conditions (37 °C, 95% air and 5% CO_2_) for 3 h. For LPS treatment, the cells were exposed to LPS after 1 h of LAM or PIC treatment, followed by incubation under normal culture conditions for 24 h. Then, the BV2 cells were randomly divided into the following groups: (i) normal control group (control group), (ii) LAM-pretreated control group (control + LAM group), (iii) OGD/R or LPS group, (iv) LAM-pretreated OGD/R or LAM-pretreated LPS group (LAM + OGD/R or LPS group), (v) PIC-pretreated control group (control + PIC group), and (vi) PIC-pretreated OGD/R or LPS group (PIC + OGD/R or LPS group).

### RNA interference experiment

The siRNA gene silencing in BV2 cells was performed as previously described [32]. Briefly, BV2 cells were seeded into a 6-well plate (Corning Inc.) at a density of 1.5 × 10^5^ cells/well 24 h prior to transfection. BV2 cells were transfected by Lipofectamine 2000 (Invitrogen; Thermo Fisher Scientific, Inc.) with siRNAs targeting to Dectin-1 (sense: 5′-GGGAAGAGCUGUUACCUAUTT-3′; antisense: 5′-AUAGGUAACAGCUCUUCCCTT-3′), Syk (sense: 5′-CCUGCUGCACGAAAGGGAAATT-3′; antisense: 5′-UUUCCCUUCGUGCAGCAGGTT-3′) or control siRNA (sense: 5′-UUCUCCGAACGUGUCACGUTT-3′; antisense: 5′-ACGUGACACGUUCGGAGAATT-3′) as the negative control. Two days after transfection, the knockdown efficiency of siRNA was determined via western blot analysis. The present study assigned the cells into the following groups: (i) control siRNA group, (ii) Dectin-1 or Syk siRNA group, (iii) OGD/R + control siRNA group, and (iv) OGD/R + Dectin-1 or Syk siRNA group.

### Western blotting

Total proteins from the ischemic cortical area brain tissues or BV2 microglial cells were collected in equal amounts of cell lysate, and the separated proteins in the supernatant were subsequently transferred. For immunoblotting, the following primary antibodies were used: Anti-Dectin-1 (1:1,000; Abcam), anti-Syk (1:1,000; Cell Signaling Technology, Inc.), anti-p-Syk (1:1,000; Cell Signaling Technology, Inc.), anti-TNF-α (1:1,000; Cell Signaling Technology, Inc.), and anti-iNOS (1:1,000; Cell Signaling Technology, Inc.). Western blotting was performed at 6 h and 3, 5, and 7 days after ischemic stroke for the mouse tissues and at 0, 3, 6, and 12 h after OGD/R treatment and 3, 6, 12, and 24 h after LPS treatment for the BV2 microglial cells. Then, the optimal time points of 3 days for the in vivo experiments and 3 h for OGD/R treatment and 24 h for LPS treatment in vitro were selected for the subsequent experiments.

### Immunofluorescence analysis

Brain tissue was removed and then immersed in 4% paraformaldehyde for 5 h and 20% sucrose for 3 days at 4 °C to process the samples for immunofluorescence. Following standard histological procedures, the sections were preincubated with 5% goat serum at room temperature for 60 min. Subsequently, the sections were incubated overnight at 4 °C in a mixture containing a rabbit polyclonal anti-Iba1 antibody (1:500; Abcam) and a rat polyclonal anti-CD68 antibody (1:500; Abcam). After washing three times in PBS for 10 min per wash, the sections were incubated with a fluorescent secondary immunoglobulin G antibody (polyclonal anti-rabbit Alexa Fluor 594-conjugated red antibody; 1:1,000; ProteinTech Group, Inc.) for 2 h at room temperature. After washing three times in PBS for 10 min per wash, the sections were stained with DAPI, sealed and mounted, and imaged using a fluorescence microscope (Olympus Corporation)*.* The numbers of target cells were counted using the ImageJ software.

### Statistical analysis

The data are presented as the mean ± standard deviation from three independent experiments. Comparisons between groups were performed using one-way ANOVA followed by the Student-Newman-Keuls test, and changes in the behavioral responses to drug stimuli over time among groups were tested using two-way ANOVA with repeated measures followed by the Bonferroni post hoc test. The statistical significance of differences was analyzed with the SPSS 19.0 software (IBM Corp.). Images were created using GraphPad Prism 6.0 software (GraphPad Software, Inc.). *P* < 0.05 was considered to indicate a statistically significant difference.

## Results

### Dectin-1 is significantly increased in the ischemic brain tissue after stroke and the BV2 microglial cells after OGD/R-induced injury in vitro

**Fig. 2:**
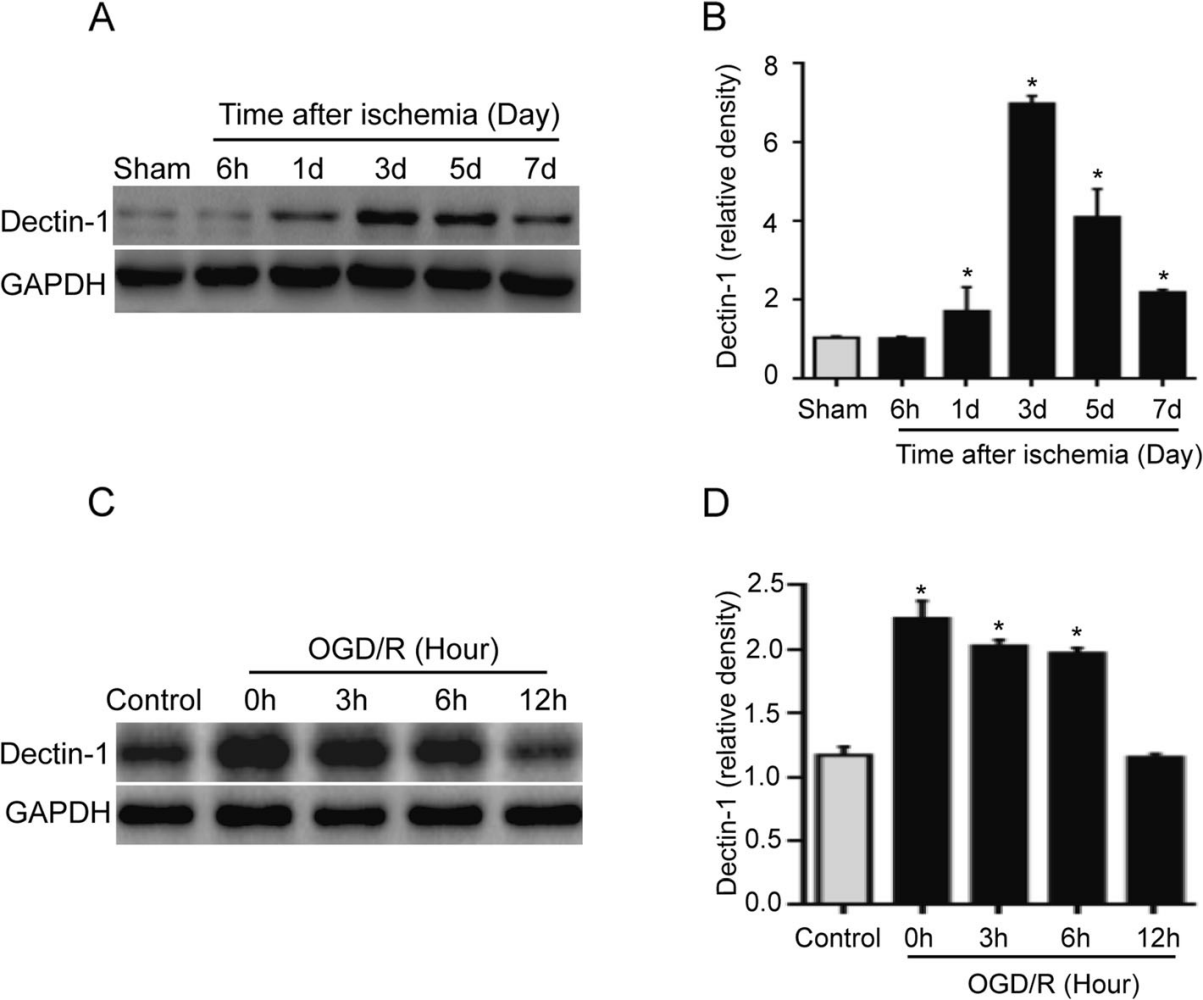
Dectin-1 expression was increased in ischemic brain tissue after a stroke and BV2 cells with OGD/R-induced injury in vitro. **a**, **b** The Dectin-1 expression level was significantly higher on days 1, 3, 5, and 7 after stroke in the ischemic group compared with in the sham group (*n* = 3/group; ^*^P < 0.05 vs. sham group). **c**, **d** The Dectin-1 expression level was significantly higher in BV2 cells with OGD/R exposure for 0, 3, and 6 h than in those without OGD/R exposure (*n* = 3/group; ^*^P < 0.05 vs. control group)

In order to investigate the potential role of Dectin-1 in the progression of ischemic stroke, the present study first examined the Dectin-1 protein levels in ischemic brain tissue and BV2 cells in the OGD/R model. Figure 2a, b demonstrates that the Dectin-1 expression level was significantly higher in the ischemic group than in the sham group; the expression level increased significantly at day 1, peaked at day 3, and decreased at day 5 after ischemic stroke (*n* = 3/group; *P* < 0.05). Figure 2c, d demonstrates that the Dectin-1 expression level was higher in the BV2 cells after 3-h OGD followed by 0, 3, and 6 h of reperfusion in the OGD/R group compared with in the control group cells (*n* = 3/group; *P* < 0.05). These results demonstrate increased Dectin-1 expression in response to a stroke.

### Syk and p-Syk are significantly upregulated in the ischemic brain tissue after stroke and BV2 microglial cells with OGD/R-induced injury in vitro

**Fig. 3:**
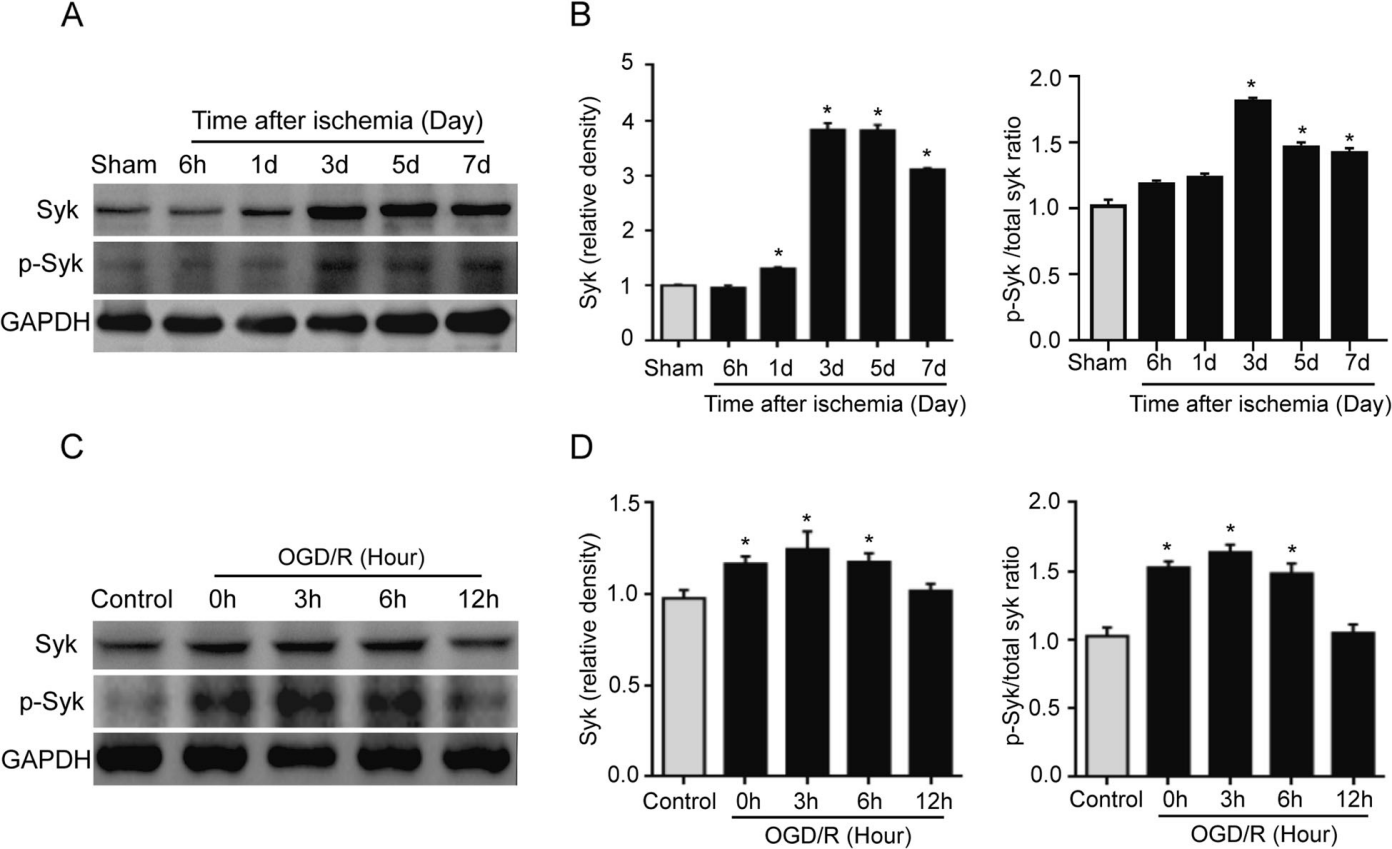
Syk and p-Syk protein expression was significantly increased in ischemic brain tissue and OGD/R-treated BV2 cells. **a**, **b** Syk and p-Syk expression was significantly enhanced on days 1, 3, 5, and 7 after a stroke in the ischemic group compared with that of the sham group (*n* = 3/group; ^*^P < 0.05 vs. sham group). **c**, **d** BV2 cells with OGD/R exposure for 0, 3, and 6 h showed significantly enhanced Syk and p-Syk expression (*n* = 3/group; ^*^P < 0.05 vs. control group)

In order to investigate whether Syk signaling was involved in the progression of ischemic stroke, the Syk and p-Syk protein levels were tested in ischemic brain tissue and BV2 cells in the OGD/R model in the present study. Figure 3a, b demonstrates that the Syk expression was significantly increased at days 1, 3, 5, and 7 after the stroke in the ischemic group compared with that of the sham group (*n* = 3/group; *P* < 0.05). Additionally, p-Syk expression was significantly enhanced at days 3, 5, and 7 after the stroke. Figure 3c, d shows that the Syk and p-Syk expression levels were higher in the BV2 cells subjected to a 3-h OGD followed by 0-, 3-, or 6-h reperfusion in the OGD/R group compared with those of the control group cells (*n* = 3/group; *P* < 0.05). Therefore, the Syk and p-Syk expression levels are enhanced following a stroke, and day 3 after a stroke in the in vivo study, and a 3-h OGD followed by a 3-h reperfusion in the in vitro study were selected for the subsequent experiments.

### Blockage of Dectin-1 rescues the brain infarct volume and neurological impairment after a stroke

**Fig. 4:**
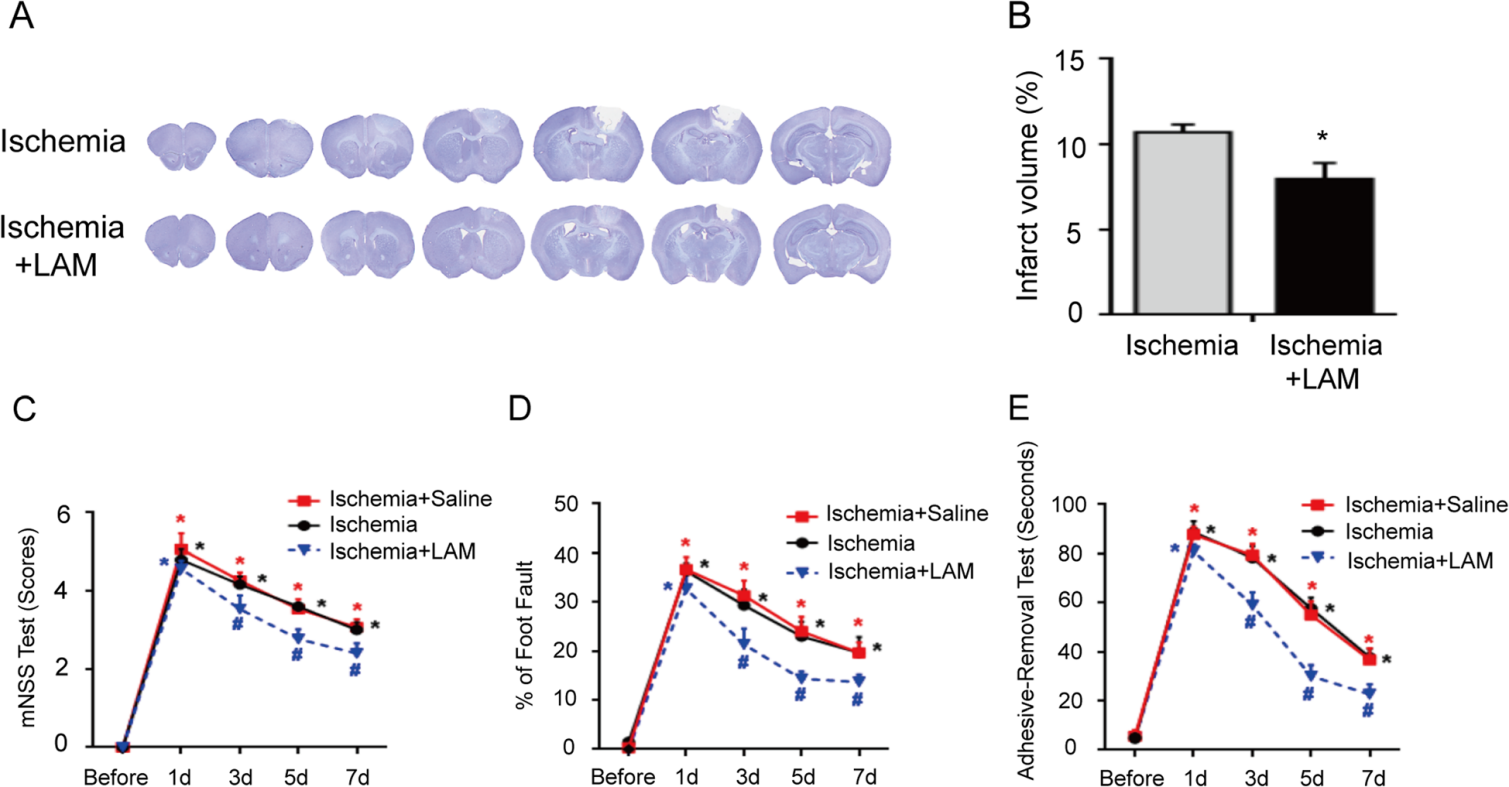
LAM treatment decreased the infarct volume and neurological impairment after a stroke. **a**, **b** LAM treatment significantly decreased the brain infarct volume in the ischemia group (*n* = 3/group; ^*^P < 0.05 vs. ischemia group). **c**–**e** LAM treatment significantly enhanced the functional outcomes on days 3, 5, and 7 after stroke in the ischemia group compared with those of the ischemia and ischemia + saline groups (*n* = 9/group; ^*^P < 0.05 vs. sham group; ^#^*P* < 0.05 vs. ischemia group/ischemia + saline group). However, no significant difference was observed between the ischemic group and ischemic + saline group on days 3, 5 and 7 after a stroke. In addition, no significant difference was observed among these three groups on day 1 after the stroke

In order to further assess the potential role of Dectin-1 in ischemic stroke, the present study examined the brain infarct volume and neurological impairment using neurological functional tests in ischemic mice with or without LAM (Dectin-1 antagonist) treatment. Figure 4a, b revealed that the infarct volume was decreased in the ischemic mice with LAM treatment compared with that of the ischemic mice without treatment (*n* = 3/group; *P* < 0.05). Figure 4c–e demonstrates that neurological function was significantly improved in the ischemic mice with LAM treatment compared with that of the ischemic mice without treatment (*n* = 9/group; *P* < 0.05). These results suggest that Dectin-1 may have a deleterious effect on the pathophysiological process of stroke.

### Blockade of Decin-1 inhibits Dectin-1/Syk signaling

**Fig. 5:**
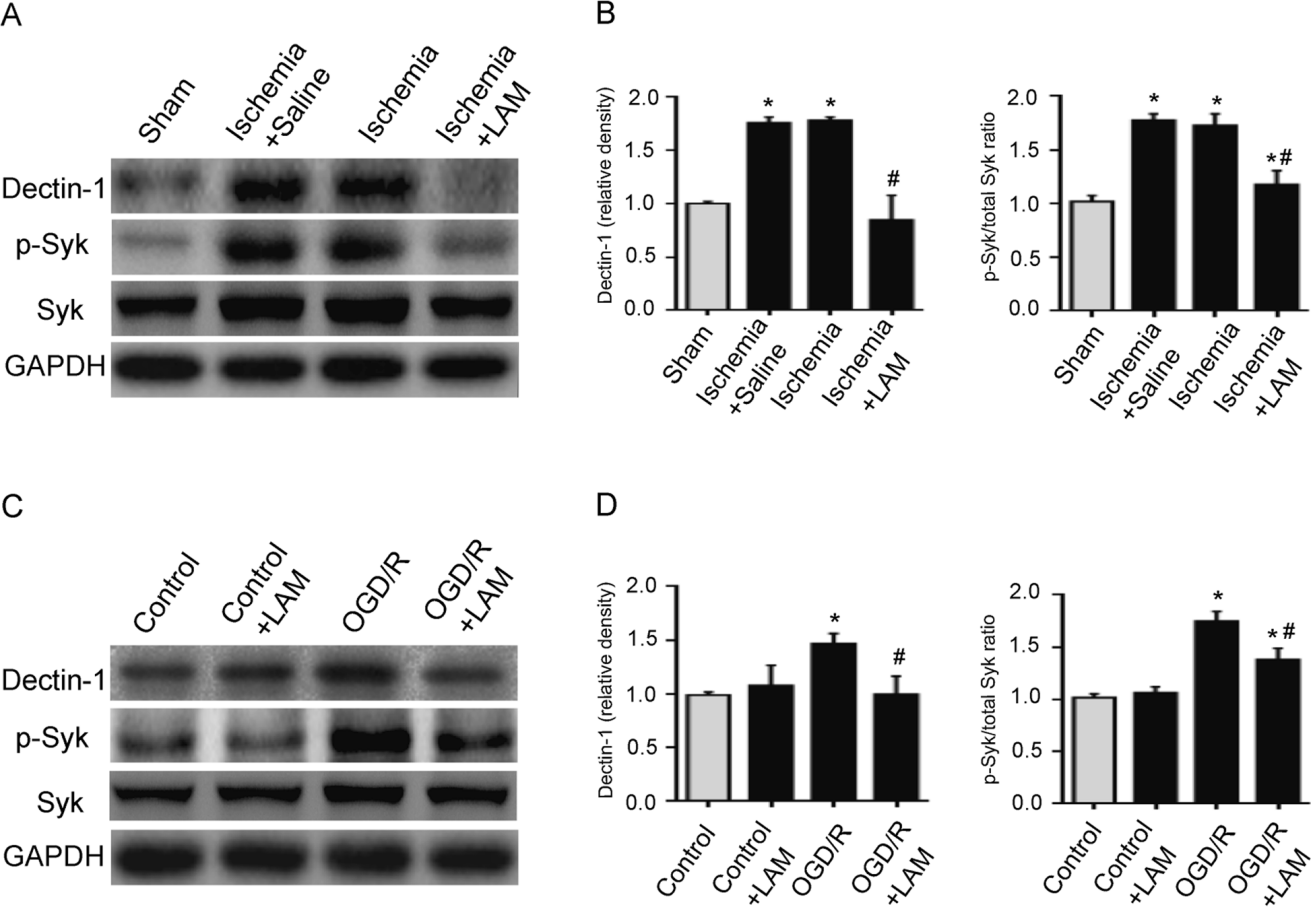
LAM treatment significantly decreased Dectin-1 and p-Syk expression. **a**, **b** Dectin-1, Syk and p-Syk expression was significantly enhanced in the brain tissue in the ischemia and ischemia + saline groups compared with that of the sham group. However, LAM treatment significantly decreased Dectin-1 and p-Syk expression in the ischemia group (*n* = 3/group; ^*^P < 0.05 vs. sham group; ^#^*P* < 0.05 vs. ischemia group/ischemia + saline group). **c**, **d** The Dectin-1, Syk, and p-Syk expression levels were significantly increased in BV2 cells subjected to OGD/R for 3 h compared with that of the BV2 cells without OGD/R exposure. However, LAM pretreatment significantly attenuated Dectin-1 and p-Syk expression in the BV2 cells subjected to the OGD/R 3 h injury model (*n* = 3/group; ^*^P < 0.05 vs. control group; ^#^*P* < 0.05 vs. OGD/R 3 h group)

In order to further assess the potential role of Dectin-1 in ischemic stroke, Dectin-1/Syk signaling was examined via western blotting. As presented in Fig. 5a, b, Dectin-1 and p-Syk expression in the ischemic brain tissue was significantly enhanced in the ischemia and ischemia + saline groups compared with that of the sham group (*n* = 3/group; *P* < 0.05). However, LAM (dectin-1 antagonist) treatment significantly decreased Dectin-1 and p-Syk expression in the ischemia group compared with that of the ischemia and the ischemia + saline groups (*n* = 3/group; *P* < 0.05). Dectin-1 and p-Syk expression was significantly increased in the BV2 cells following OGD/R exposure. LAM pretreatment notably decreased the expression of these proteins in the BV2 cells following OGD/R exposure (Fig. 5c, d; *n* = 3/group; *P* < 0.05). Taken together, these results provide evidence for the potential involvement of Dectin-1/Syk signaling in ischemic stroke.

### Blockade of Decin-1 diminishes microglial activation and decreases the expression levels of inflammatory cytokines

**Fig. 6:**
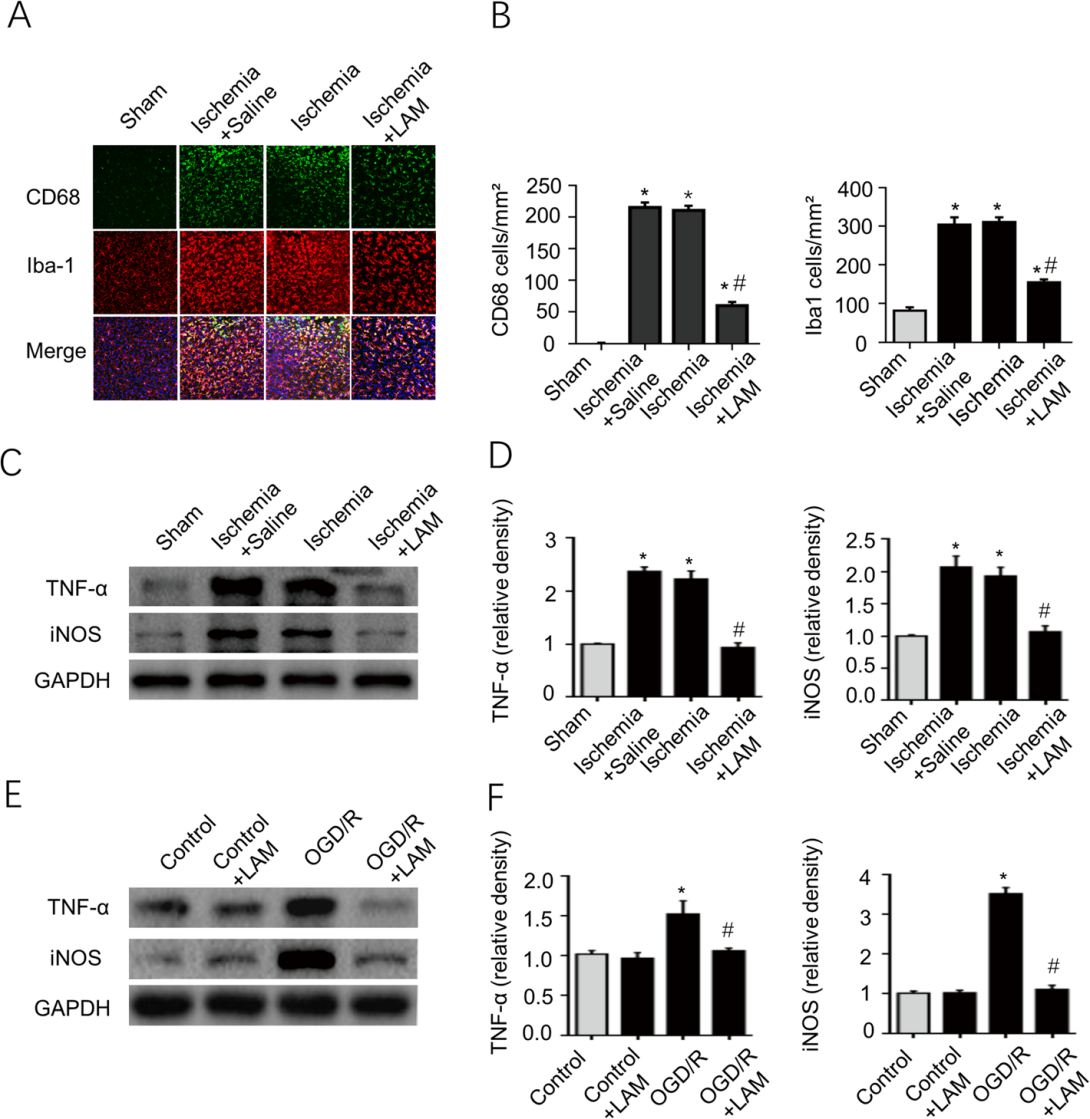
LAM treatment significantly decreased microglial activation in the ischemic mice and inhibited TNF-α and iNOS expression in the ischemic brain tissue and OGD/R-induced BV2 cells. **a**, **b** Microglial activation was assessed by Iba-1 and CD68 staining, and Iba-1- and CD68-positive cells were significantly increased in the ischemia and ischemia + saline groups. LAM treatment significantly attenuated microglial activation (scale bar, 50 μm; *n* = 3/group; ^*^P < 0.05 vs. sham group; ^#^*P* < 0.05 vs. ischemia group/ischemia + saline group). **c**, **d** TNF-α and iNOS expression was significantly enhanced in the ischemia and ischemia + saline groups compared with that of the sham group, and LAM treatment significantly inhibited TNF-α and iNOS expression in the ischemia group compared with that of the ischemia and ischemia + saline groups (*n* = 3/group; ^*^P < 0.05 vs. sham group; ^#^*P* < 0.05 vs. ischemia group/ischemia + saline group). However, no significant difference was observed between the ischemia group and ischemia + saline group. **e**, **f** TNF-α and iNOS expression was significantly increased in BV2 cells subjected to OGD/R for 3 h compared with that of cells without OGD/R exposure. LAM pretreatment of BV2 cells with OGD/R exposure significantly decreased the expression of the aforementioned proteins (*n* = 3/group; ^*^P < 0.05 vs. control group; ^#^*P* < 0.05 vs. OGD/R group).

In order to further investigate the potential role of Dectin-1 in ischemic stroke, microglial activation was assessed by Iba-1 and CD68 staining, and the expression of inflammatory cytokines was examined via western blotting. 3 days after the stroke, Iba-1- and CD68-positive cells were detected in the ischemic brain tissue, although the number of these cells was largely decreased after LAM (dectin-1 antagonist) treatment (Fig. 6a, b). Figure 6c, d demonstrates that TNF-α and iNOS expression was significantly enhanced in the ischemia and ischemia + saline groups compared with that of the sham group (*n* = 3/group; *P* < 0.05). LAM treatment significantly decreased TNF-α and iNOS expression in the ischemia group compared with that of the ischemia and ischemia + saline groups (*n* = 3/group; *P* < 0.05). Figure 6e, f shows similar results for BV2 cells exposed to OGD/R in the in vitro experiments. Taken together, these results suggest that Dectin-1 is involved in the inflammatory response after a stroke.

### Blockade of Syk partly decreases the brain infarct volume and improves the neurological outcomes after a stroke

**Fig. 7:**
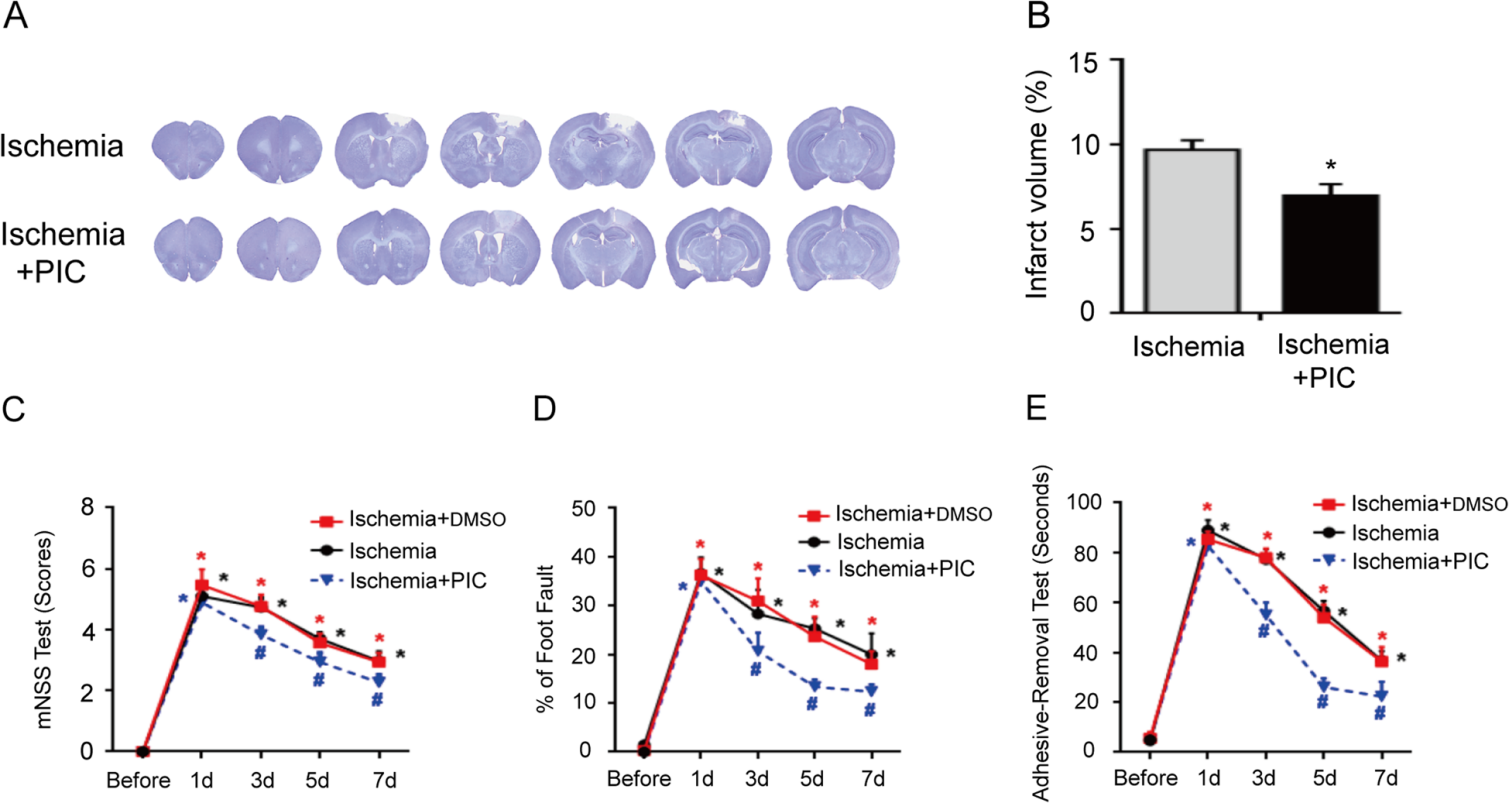
PIC treatment significantly decreased the infarct volume and neurological impairments. **a**, **b** PIC treatment significantly decreased the infarct volume in the ischemia group compared with that of the ischemia group (*n* = 3/group; ^*^P < 0.05 vs. sham group). **c**–**e** The functional impairments after stroke were significantly attenuated on days 3, 5, and 7 after PIC treatment in the ischemia group compared with those of the ischemia and ischemia + DMSO groups (*n* = 9/group; ^*^P < 0.05 vs. sham group; ^#^*P* < 0.05 vs. ischemia group/ischemia + DMSO group).

In order to further investigate whether Dectin-1/Syk signaling played a crucial role in the progression of ischemic stroke, PIC (Syk inhibitor) was applied, and the brain infarct volume and neurological functional tests were evaluated. Figure 7a, b indicated that the infarct volume was decreased in ischemic mice with PIC treatment compared with that of ischemic mice without treatment (*n* = 3/group; *P* < 0.05). Figure 7c–e shows that functional impairments after stroke were significantly attenuated at days 3, 5, and 7 after PIC treatment in the ischemic stroke model mice compared with those of the mice in the ischemia and ischemia + DMSO groups (*n* = 9/group; *P* < 0.05).

### PIC treatment attenuates p-Syk expression in ischemic brain tissue and in BV2 cells with OGD/R exposure

**Fig. 8:**
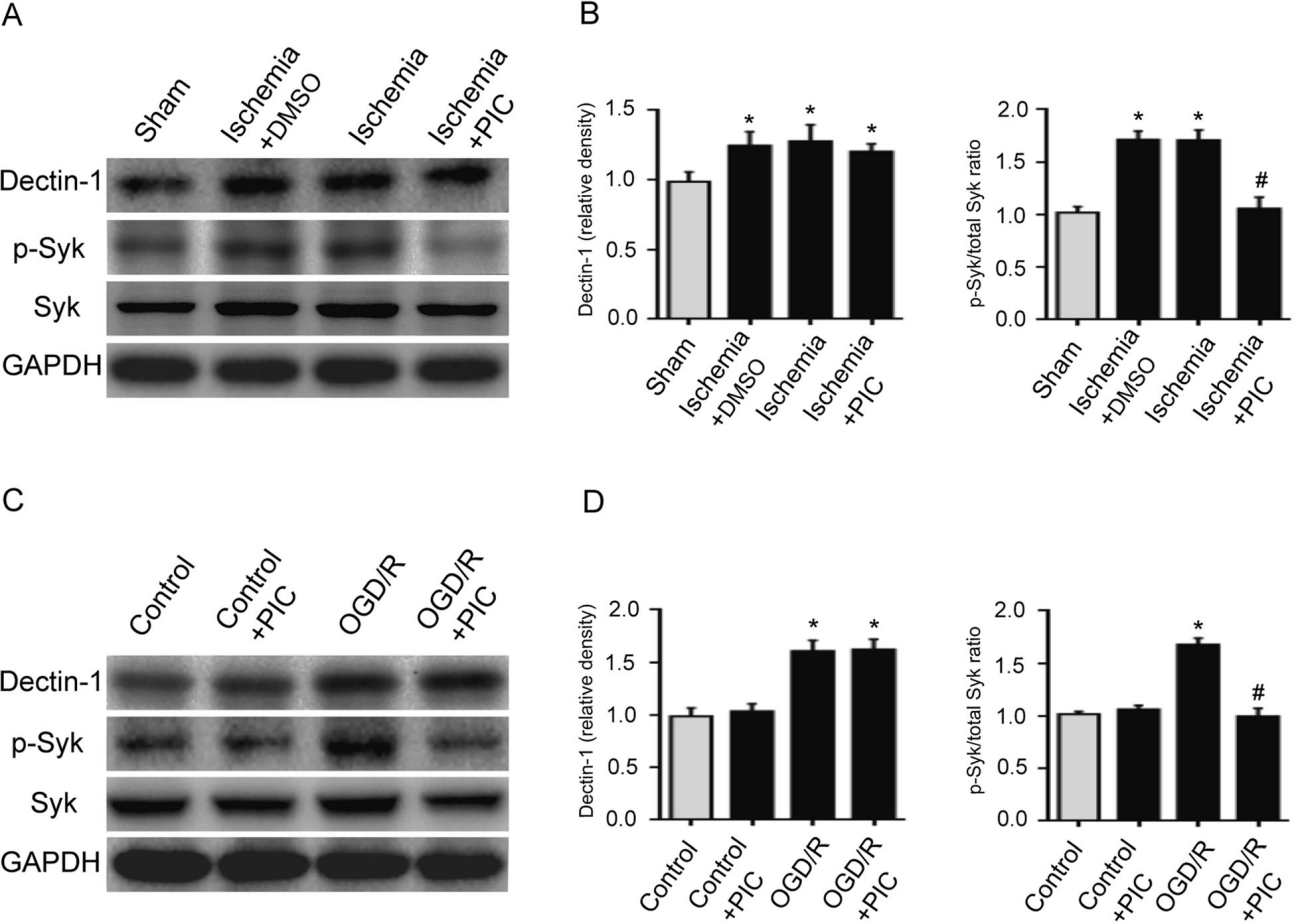
PIC treatment attenuated p-Syk expression in ischemic brain tissue and in BV2 cells with OGD/R exposure. **a**, **b** p-Syk expression was significantly enhanced in the ischemic brain tissue in the ischemia and ischemia + DMSO groups compared with that of the sham group. PIC treatment significantly decreased p-Syk expression in the ischemic brain tissue (*n* = 3/group; ^*^P < 0.05 vs. sham group; ^#^*P* < 0.05 vs. ischemia group/ischemia + DMSO group). However, PIC treatment failed to decrease Dectin-1 expression in the ischemic brain tissue. **c**, **d** Dectin-1 and p-Syk expression in BV2 cells with OGD/R exposure was significantly increased compared with that of the cells without OGD/R exposure. PIC pretreatment of BV2 cells with OGD/R exposure significantly decreased p-Syk expression (*n* = 3/group; ^*^P < 0.05 vs. control group; ^#^*P* < 0.05 vs. OGD/R group). However, Dectin-1 expression was not attenuated in the OGD/R-induced BV2 cells with PIC treatment compared with that of the OGD/R-induced BV2 cells without PIC pretreatment.

In order to investigate whether Dectin-1/Syk signaling played a crucial role in the progression of ischemic stroke, PIC (Syk inhibitor) was applied, and Dectin-1/Syk signaling was examined via western blotting in the present study. Figure 8a, b shows that p-Syk expression was significantly enhanced in the ischemic brain tissue in the ischemia and ischemia + DMSO groups compared with that of the sham group. PIC treatment significantly decreased p-Syk expression in the ischemic brain tissue (*n* = 3/group; ^*^P < 0.05 vs. sham group; ^#^*P* < 0.05 vs. ischemia group/ischemia + DMSO group). However, PIC treatment failed to decrease Dectin-1 expression in the ischemic brain tissue. Figure 8c, d demonstrates that Dectin-1 and p-Syk expression in BV2 cells with OGD/R exposure was significantly increased compared with that of the cells without OGD/R exposure. PIC pretreatment of BV2 cells with OGD/R exposure significantly decreased p-Syk expression (*n* = 3/group; ^*^P < 0.05 vs. control group; ^#^*P* < 0.05 vs. OGD/R group). However, Dectin-1 expression was not attenuated in the OGD/R-induced BV2 cells with PIC treatment compared with that of the OGD/R-induced BV2 cells without PIC pretreatment.

### Decrease of microglial activation and inflammatory cytokine expression by inhibition of Syk signaling

**Fig. 9:**
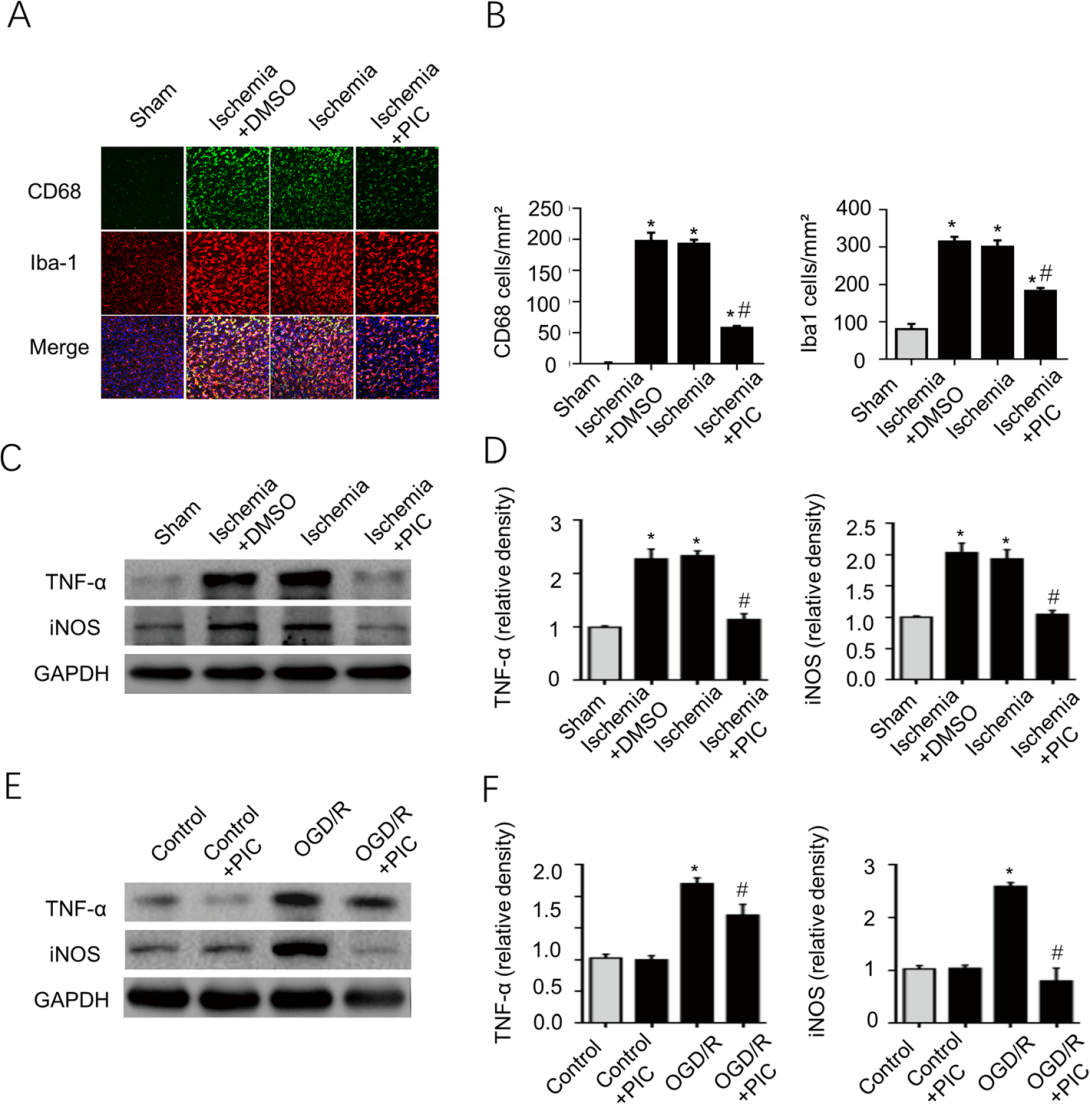
PIC treatment inhibited microglial activation and decreased TNF-α and iNOS expression in the ischemic brain tissue and BV2 cells with OGD/R exposure. **a**, **b** Iba-1 and CD68 staining showed that microglial activation was significantly enhanced in the ischemic brain tissue 3 days after stroke compared with that of the brain tissue in the sham group. PIC treatment significantly attenuated microglial activation in the brain tissue (scale bar, 50 μm; *n* = 3/group; ^*^P < 0.05 vs. sham group; ^#^*P* < 0.05 vs. ischemia group/ischemia + DMSO group). However, no significant difference was noted between the ischemic and DMSO groups. **c**, **d** TNF-α and iNOS expression was significantly enhanced in the ischemic brain tissue at 3 days after stroke compared with that of the brain tissue in the sham group. PIC treatment significantly decreased TNF-α and iNOS expression in the ischemic brain tissue (*n* = 3/group; ^*^P < 0.05 vs. sham group; ^#^*P* < 0.05 vs. ischemia group/ischemia + DMSO group). **e**, **f** BV2 cells with OGD/R exposure exhibited significantly increased TNF-α and iNOS expression compared with that of BV2 cells without OGD/R exposure. PIC pretreatment significantly inhibited TNF-α and iNOS expression in BV2 cells with OGD/R exposure compared with that of the OGD/R group (*n* = 3/group; ^*^P < 0.05 vs. control group; ^#^*P* < 0.05 vs. OGD/R group). However, PIC pretreatment failed to decrease TNF-α and iNOS expression in BV2 cells without OGD/R exposure

To further study the role of Dectin-1/Syk signaling in ischemic stroke, the Syk inhibitor PIC was used in the present study. Figure 9a, b shows that PIC treatment significantly decreased the numbers of Iba-1- and CD68-positive cells in the ischemic brain tissue of the ischemia group compared with that of the ischemia and ischemia + DMSO groups (*n* = 3/group; *P* < 0.05). Figure 9c, d demonstrates that TNF-α and iNOS expression was significantly decreased in the ischemic brain tissue in the ischemia + PIC group compared with that of the ischemia and ischemia + DMSO groups (*n* = 3/group; *P* < 0.05). Figure 9e, f showed similar results for the BV2 cells with OGD/R exposure in the in vitro experiments. Taken together, these results suggest the involvement of Dectin-1/Syk signaling in microglial activation and enhanced inflammatory cytokine expression after stroke.

### LAM and PIC decreases TNF-α and iNOS production by inhibiting Dectin-1/Syk signaling in primary microglial cells with OGD/R-induced injury in vitro

**Fig. 10:**
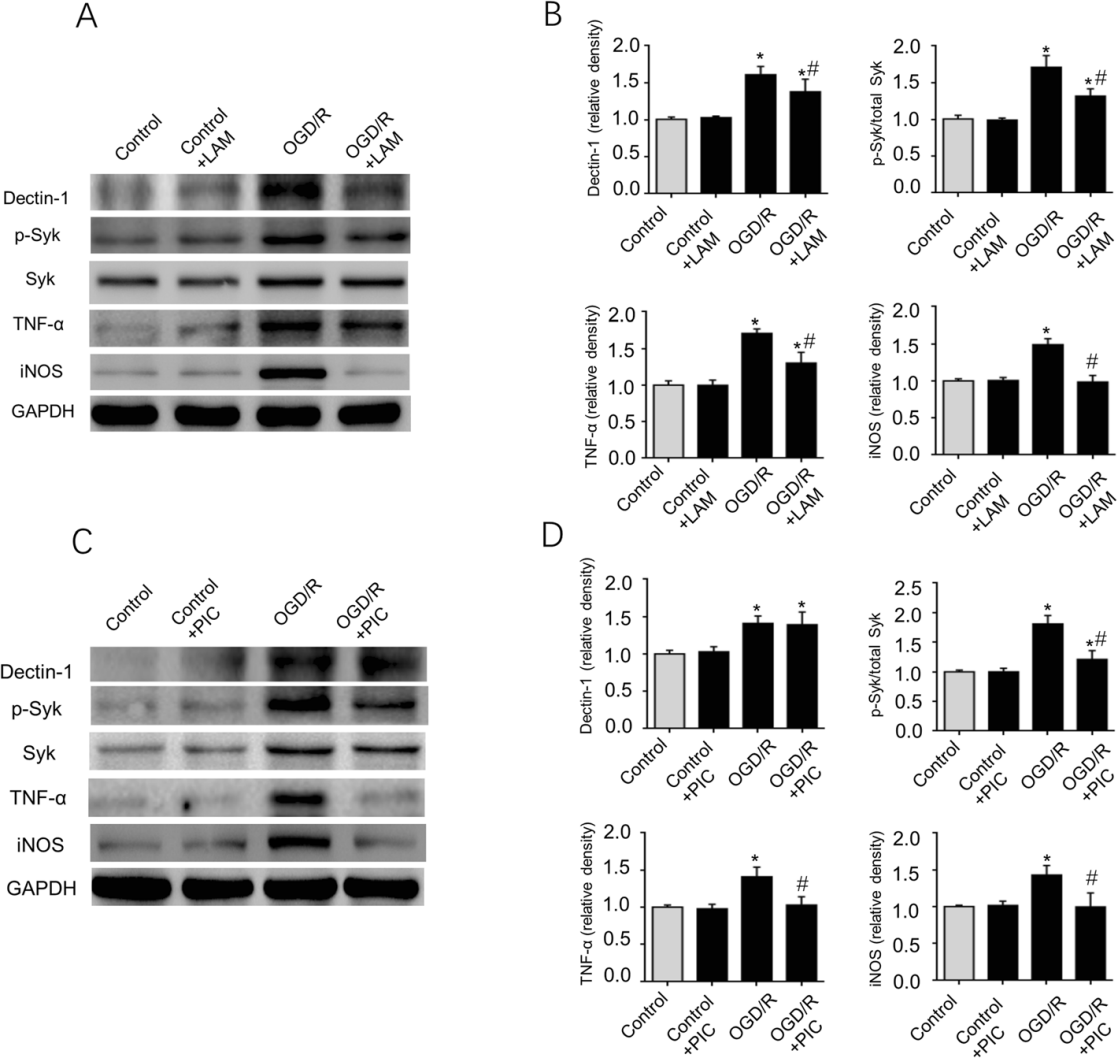
Dectin-1, Syk and p-Syk expression was significantly increased in primary microglial cells with OGD/R exposure. LAM/PIC pretreatment significantly decreased p-Syk, TNF-α, and iNOS expression in primary microglial cells following OGD/R exposure. **a**, **b** Dectin-1, p-Syk, TNF-α, and iNOS expression levels were significantly enhanced in primary microglial cells with OGD/R exposure compared with primary microglial cells without OGD/R exposure. LAM pretreatment significantly decreased the expression levels of Dectin-1, p-Syk, TNF-α, and iNOS in primary microglial cells with OGD/R exposure (*n* = 3/group; ^*^P < 0.05 vs. control group; ^#^*P* < 0.05 vs. OGD/R group). **c**, **d** PIC pretreatment significantly inhibited p-Syk, TNF-α, and iNOS expression in primary microglial cells with OGD/R exposure compared with cells without OGD/R exposure (*n* = 3/group; ^*^P < 0.05 vs. control group; ^#^*P* < 0.05 vs. OGD/R group)

The present study further investigated the proinflammatory effect of Dectin-1/Syk signaling after primary microglial cells were exposed to OGD/R-induced inflammation. As presented in Fig. 10a, b, the Dectin-1, p-Syk, TNF-α and iNOS expression levels were significantly increased in primary microglial cells following OGD/R induction. In addition, LAM pretreatment significantly decreased the Dectin-1 and p-Syk expression levels. At the same time, TNF-α and iNOS production was significantly decreased in primary microglial cells following LAM treatment (*n* = 3/group; *P* < 0.05). Figure 10c, d shows that p-Syk expression was significantly inhibited following PIC pretreatment, and PIC treatment also significantly decreased p-Syk, TNF-α, and iNOS production in the OGD/R-induced primary microglial cells (*n* = 3/group; *P* < 0.05).

### Knockdown of Dectin-1 or Syk inhibits production of proinflammatory cytokines TNF-α and iNOS in BV2 microglial cells following OGD/R stimulation

**Fig. 11:**
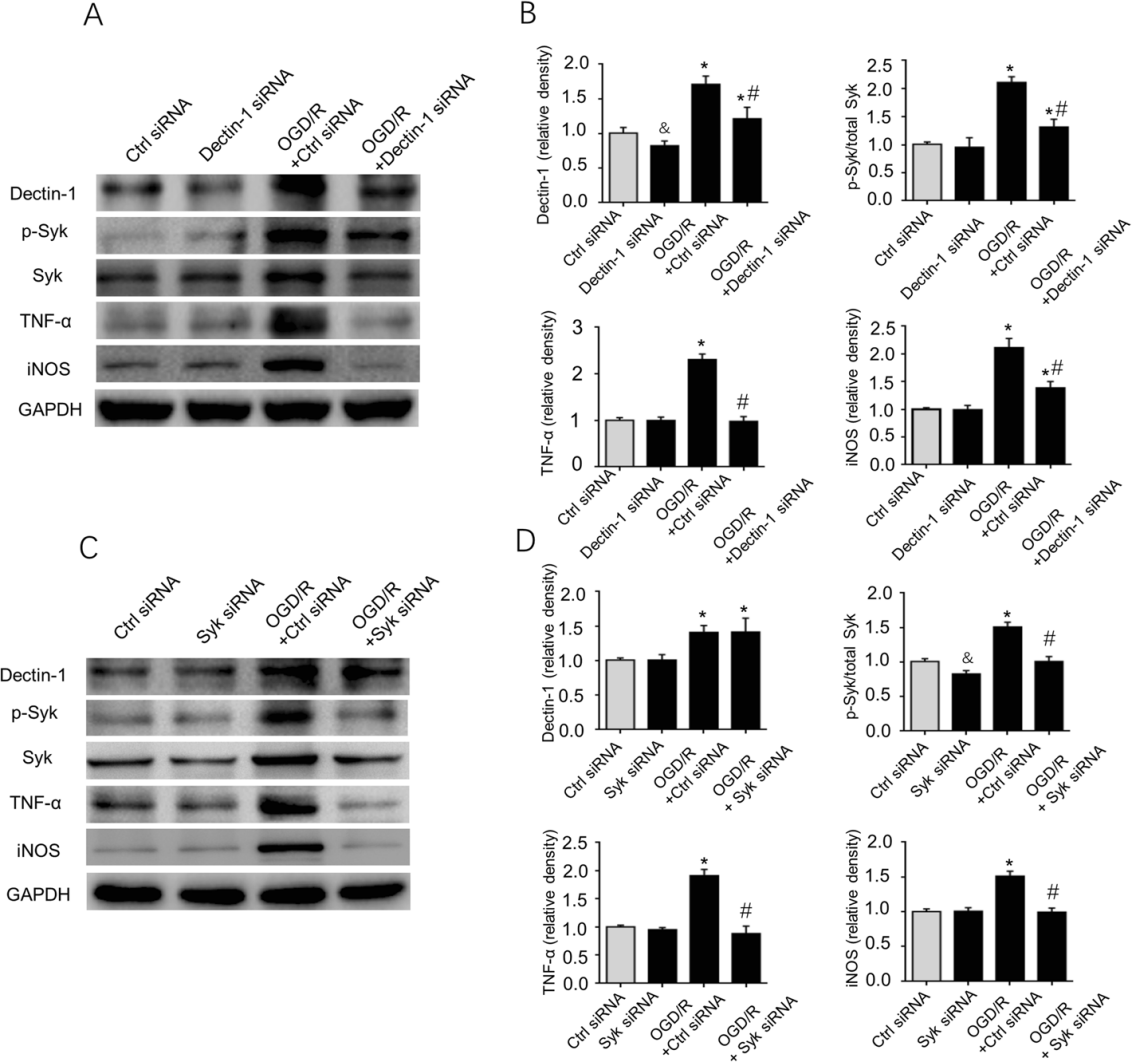
Effects of knockdown Dectin-1 or Syk on inflammatory responses in OGD/R-induced BV2 cells. **a**, **b** The Dectin-1 siRNA decreased Dectin-1 expression in BV2 cells with OGD/R exposure (*n* = 3/group; ^&^*P* < 0.05 vs. control siRNA group). The expression levels of p-Syk, TNF-α, and iNOS were also downregulated in OGD/R + Dectin-1 siRNA group compared with that of the BV2 cells in OGD/R + control siRNA group (*n* = 3/group; ^*^P < 0.05 vs. control siRNA group; ^#^*P* < 0.05 vs. OGD/R + control siRNA group). **c**, **d** The Syk siRNA decreased Syk expression in BV2 cells (*n* = 3/group; ^&^*P* < 0.05 vs. control siRNA group). Knockdown Syk also attenuated the production of p-Syk, TNF-α, and iNOS in BV2 cells in the OGD/R + Syk siRNA group compared with the OGD/R + control siRNA group (*n* = 3/group; ^*^P < 0.05 vs. control siRNA group; ^#^*P* < 0.05 vs. OGD/R + control siRNA group)

In order to further precisely evaluate the function of Dectin-1/Syk signaling to regulate TNF-α and iNOS, BV2 cells were stimulated with OGD/R following transfection with Dectin-1 siRNA or Syk siRNA. Figure 11a, b shows that knockdown of Dectin-1 downregulated the expression of p-Syk, TNF-α, and iNOS in BV2 cells in the OGD/R + Dectin-1 siRNA group compared in the OGD/R + control siRNA group (*n* = 3/group; *P* < 0.05). Similarly, it was revealed that the production of p-Syk, TNF-α, and iNOS in BV2 cells was significantly decreased in the OGD/R + Syk siRNA group compared in the OGD/R + control siRNA group. The present study also demonstrated that Dectin-1 expression was significantly decreased in BV2 cells in the Dectin-1 siRNA group compared in the control siRNA group, and the Syk expression was decreased in BV2 cells in Syk siRNA group compared in the control siRNA group (*n* = 3/group; *P* < 0.05). These data suggested that knockdown of Dectin-1 or Syk ameliorate inflammation progression in BV2 microglial cells with OGD/R treatment.

### LAM and PIC decreases TNF-α and iNOS production by inhibiting Dectin-1/Syk signaling in BV2 microglial cells with LPS-induced injury in vitro

**Fig. 12:**
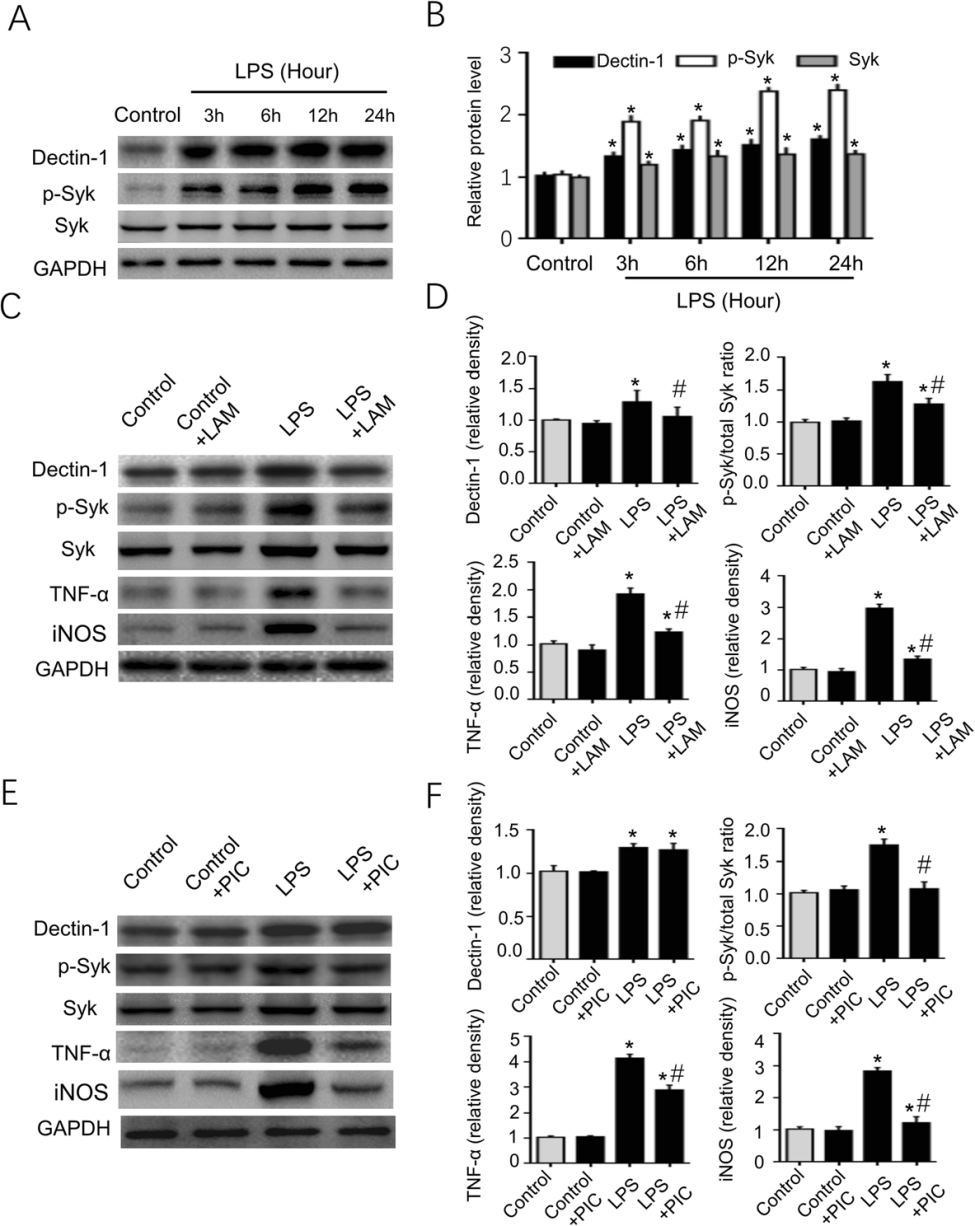
Dectin-1, Syk, and p-Syk expression levels are significantly increased in LPS-induced BV2 cells. LAM/PIC pretreatment significantly decreased p-Syk, TNF-α, and iNOS expression in BV2 cells following LPS exposure. In addition, Dectin-1 expression was significantly decreased in the LPS-induced BV2 cells following LAM pretreatment. **a**, **b** The Dectin-1 and p-Syk expression levels were significantly higher at 3, 6, 12, and 24 h in the BV2 cells following LPS exposure compared with in the BV2 cells without LPS exposure (*n* = 3/group; ^*^P < 0.05 vs. control group). Therefore, 24 h was selected as the optimal time point for BV2 cells exposed to LPS. **c**, **d** Dectin-1, p-Syk, TNF-α, and iNOS expression was significantly enhanced in BV2 cells with 24 h of LPS exposure compared with that of BV2 cells without LPS exposure. LAM pretreatment significantly decreased the expression levels of the aforementioned proteins in BV2 cells with 24 h of LPS exposure (*n* = 3/group; ^*^P < 0.05 vs. control group; ^#^*P* < 0.05 vs. LPS group). **e**, **f** BV2 cells with LPS exposure demonstrated significantly increased Dectin-1, p-Syk, TNF-α, and iNOS expression levels compared with that of BV2 cells without LPS exposure. PIC pretreatment significantly inhibited Syk, TNF-α, and iNOS expression in BV2 cells with LPS exposure compared with that of the LPS group (*n* = 3/group; ^*^P < 0.05 vs. control group; ^#^*P* < 0.05 vs. LPS group). However, PIC pretreatment failed to decrease Dectin-1 expression in BV2 cells with PIC pretreatment

In order to further investigate the proinflammatory effect of Dectin-1/Syk signaling in brain tissue after a stroke, BV2 cells were exposed to LPS-induced inflammation. As presented in Fig. 12a, b, the Dectin-1 and p-Syk expression levels were significantly increased in the BV2 cells at 3, 6, 12, and 24 h after LPS induction. The 24 h was selected as the LPS induction time point in BV2 cells in the subsequent experiments. Figure 12c, d shows that LAM pretreatment significantly decreased the Dectin-1 and p-Syk expression levels. At the same time, TNF-α and iNOS production was significantly decreased in the BV2 cells after LAM treatment. Figure 12e, f shows that p-Syk expression was significantly inhibited following PIC pretreatment, but no significant difference in Dectin-1 expression was observed between the LPS and LPS + PIC groups. PIC treatment also significantly decreased TNF-α and iNOS production in the LPS-induced BV2 cells.

## Discussion

The present study demonstrated that the Dectin-1, Syk, p-Syk, TNF-α, and iNOS expression levels were significantly increased in ischemic brain tissue after a stroke. Either Dectin-1 antagonist (LAM) or Syk inhibitor (PIC) treatment significantly decreased the expression levels of these proteins in the ischemic brain tissue. At the same time, the present study also revealed that LAM or PIC treatment significantly decreased the infarct volume and improved the functional outcomes after a stroke. Similar results were observed in the in vitro experiments. Taken together, the in vivo and complementary in vitro data support the hypothesis that Dectin-1/Syk signaling plays a vital role in neuroinflammation after a stroke.

Microglia are the resident mononuclear phagocytic cells that are critical for inflammatory responses in the central nervous system (CNS). The most common distinguishing feature of the microglia is their rapid activation in response to pathological changes in the CNS, such as ischemia [33]. Dectin-1 is expressed at a low level in the brain but is strongly upregulated following exposure to various stimuli, such as ischemia and injury [34, 35]. Dectin-1 has been reported to activate the NLRP3 (NLR family, pyrin domain-containing 3) inflammasome and enhance IL-1β production [36–38]. Activation of Dectin-1 can cause macrophage-mediated demyelination and axonal injury, and blockade of Dectin-1 can decrease inflammatory macrophage-mediated injury following spinal cord injury [39]. A low-fat diet with caloric restriction decreases the expression of Dectin-1 and then attenuates white matter microglia activation during aging [40]. It has also been demonstrated that Dectin-1 signaling can trigger neuroinflammation and enable repair of injured central nervous system neurons [41]. The present study demonstrated that Dectin-1 was significantly increased in ischemic brain tissue following a stroke, and in the OGD/R-treated BV2 cells and primary microglia. The number of activated microglia were also enhanced after a stroke. Dectin-1 antagonist (LAM) treatment significantly decreased the expression of the aforementioned proteins and attenuated the number of activated microglia. It was also revealed that the blockade of Dectin-1 attenuated the brain infarct volume and decreased neurological deficits and microglial activation after a stroke. These data suggest that Dectin-1 overexpression may exert deleterious effects on the brain tissue and enhance neuroinflammation following ischemic stroke.

Syk, highly expressed in the microglia, plays a vital role in the inflammatory responses after ischemic stroke [42]. The ligands produced by necrotic cells bind to the receptor of Dectin-1, resulting in recruitment and activation of Syk [43–45]. Syk inhibitor PIC decreases neuronal damage after retinal ischemia-reperfusion injury [46, 47]. Previous studies have demonstrated that Dectin-1 can activate Syk-dependent intracellular signaling cascades [48]. Furthermore, the Dectin-1/Syk signaling pathway is involved in ROS generation and NLRP3 activation in response to β-glucan particles [36, 49, 50]. Activated Syk (p-Syk) leads to subsequent activation of downstream signaling molecules, such as p85, PKB, PDK1, and NF-κB, resulting in the expression of proinflammatory genes, including TNF-α, COX-2, and iNOS [51–54]. The present study demonstrated that Syk and p-Syk were significantly increased in the ischemic brain tissue after a stroke, as well as in the OGD/R model. Syk inhibitor (PIC) treatment significantly attenuated p-Syk, TNF-α, and iNOS expression in the ischemic brain tissue after a stroke and in the OGD/R model. Other inflammatory mediators such as JNK, p38 MAPK, and NF-kB have been linked to microglia activation [55–60] and would be assessed in future work. The present study also revealed that the Syk inhibitor decreased microglial activation, the brain infarct volume, and neurological deficits after a stroke. These data suggest that Syk may also exert harmful effects on brain tissue and enhance neuroinflammation after an ischemic stroke.

## Conclusions

The present study demonstrated that Dectin-1, Syk, and p-Syk expression was significantly increased after a stroke both in vivo and in vitro. Treatment of ischemic stroke with a Dectin-1 antagonist or a Syk inhibitor significantly decreased microglial activation, the brain infarct volume, neurological impairment, and production of the proinflammatory cytokines TNF-α and iNOS after ischemic stroke. The present study may offer new ideas for effective treatment of patients who have suffered from a stroke. Further studies of the pathophysiological functions of Dectin-1/Syk signaling in the activated inflammatory response may prove beneficial for clinical applications.

## Supplementary information

**Additional file 1.** The optimal dose for LAM and PIC in BV2 cells.

## Data Availability

The datasets used and/or analyzed during the present study are available from the corresponding author upon reasonable request.

## Abbreviations

Dectin-1: Dendritic cell-associated C-type lectin-1
Syk: Spleen tyrosine kinase
p-Syk: Phosphorylated spleen tyrosine kinase
TNF-α: Tumor necrosis factor-α
iNOS: Inducible nitric oxide synthase
OGD/R: Oxygen-glucose deprivation/reoxygenation
LAM: Laminarin
PIC: Piceatannol
LPS: Lipopolysaccharide
DC: Dendritic cell
ITAM: Immune-receptor tyrosine-based activation motif
CLR: C-type lectin receptor
SH2: Src homology-2 domains
CLEC2: C-type lectin-like receptor 2
CLEC9A: C-type lectin domain-containing 9A
DMSO: Dimethyl sulfoxide
mNSS: Modified neurological severity score
DMEM: Dulbecco’s modified Eagle’s medium
PBS: Phosphate-buffered saline
PKB: Protein kinase B
PDK1: Phosphoinositide-dependent protein kinase-1
NF-κB: Nuclear factor kappa B
COX-2: Cyclooxygenase-2
ROS: Reactive oxygen species
NLRP3: NLR family pyrin domain-containing 3
DAMPs: Damage-associated molecular patterns
