# Supplementary figures and images for: Dectin-1/Syk signaling triggers neuroinflammation after ischemic stroke in mice
Source: J Neuroinflammation. 2020 Jan 11;17:17. doi: 10.1186/s12974-019-1693-z (PMC6954534; doi:10.1186/s12974-019-1693-z)

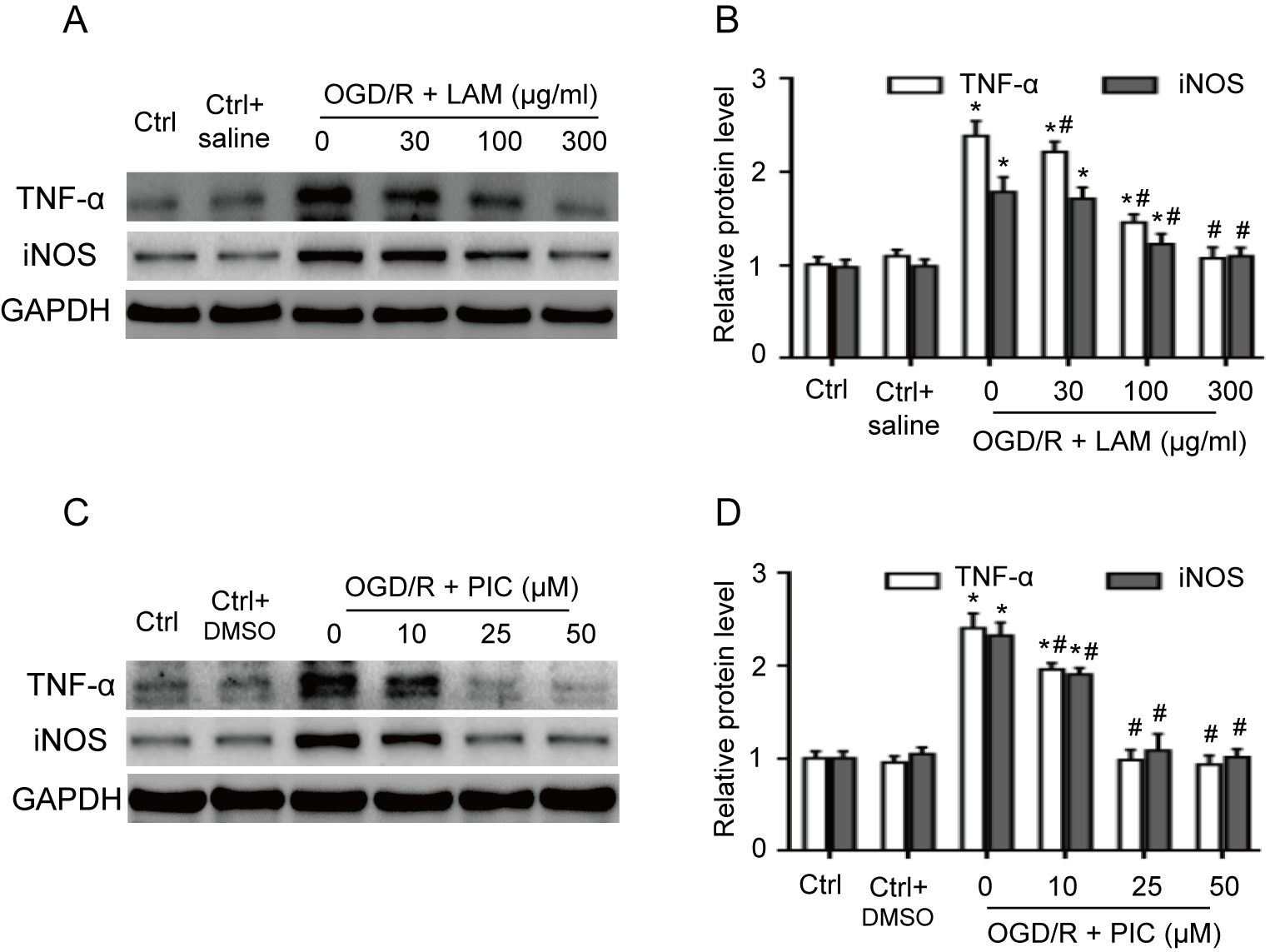

Supplement: Supplementary file 1 — Additional file 1. The optimal dose for LAM and PIC in BV2 cells. [file 12974_2019_1693_MOESM1_ESM.tif]
